# Supplementary material for: Knowledge of female genital cutting among health and social care professionals in Francophone Belgium: A cross-sectional survey
Source: PLOS Glob Public Health. 2024 Jul 18;4(7):e0002225. doi: 10.1371/journal.pgph.0002225 (PMC11257331; doi:10.1371/journal.pgph.0002225)
Supplement: S1 Text — (PDF) [file pgph.0002225.s001.pdf]

Version test differences Belgique – France
Knowledge, Attitudes and Practice (KAP) - RHCforFGC - Gender Net
Version: 1 avril 2021

Prévenir, repérer et répondre aux besoins des filles et des femmes vivant avec une excision ou à risque d'excision

---

1. **Quelle est votre profession?**

☐ Médecin
Merci de préciser votre spécialité:
  ☐ Gynécologue/obstétricen.ne
  ☐ Médecin généraliste
  ☐ Psychiatre
  ☐ Pédiatre
  ☐ Médecine des voyage
  ☐ Infectiologue
  ☐ Urgentiste
  ☐ Anesthésiste-réanimateur / Intensiviste
  ☐ Autre:

- Merci de préciser votre statut :
  ☐ Interne / Médecin assistant
  En quelle année ?: …..
  ☐ Médecin titulaire
  ☐ Docteur.e en médecine

☐ Sage-femme
☐ Etudiant.e sage-femme
☐ Infirmier.e
☐ Etudiant.e infirmier.e
☐ Travailleur.se social.e
☐ Etudiant.e d'assistance sociale
☐ Médiateur/trice en santé
☐ Sexologue
☐ Psychologue
☐ Etudiant.e psychologue
☐ Autre (merci de préciser) :

2. **Dans quel(s) service(s) travaillez-vous (plusieurs réponses possible)**

☐ Consultation pré-natale
☐ Salle d'accouchement

☐ Service de suites de couches/post-partum
☐ Maison de naissance
☐ Service de pédiatrie
☐ Service de médecine interne
☐ Service de chirurgie
☐ Service de médecine du voyage / Travel Clinic
☐ Maison médicale
☐ Centre de planning familial / Centre de santé spécifique à la santé des femmes
☐ Centre d'accueil pour demandeurs d'asile (Fedasil, Croix-Rouge, Rode Kruis, Caritas,…)
☐ Office de la naissance et de l'enfance (Belgique)
☐ Service de maladies infectieuses et/ou médecine tropicale
☐ Centre de dépistage des infections sexuellement transmissibles
☐ Centre de lutte contre la tuberculose
☐ Centre Public d'Action Sociale (CPAS)
☐ Organisation non gouvernementale (ONG)/ Association sans but lucratif (asbl)
☐ Autre (merci de préciser) :……………………………………………….

Pratique professionnelle

3. Avez-vous déjà entendu parler de l'excision (tous types de sources)?

☐ Non (➔ question 4)
☐ Oui
☐ Je ne suis pas sûr.e

3.1 Si oui, à quelle(s) occasion(s) ? (plusieurs réponses possible)

☐ Formation pendant le curriculum de base (universitaire ou haute école)
☐ Formation post-universitaire type DU ou DIU
☐ Formation pratique clinique au sein du service dans lequel j'exerce
☐ Staff médicaux (revues de cas)
☐ Formation continue

Quel(s) type(s) de formation :

a. Mooc, e-learning
b. Séances en présentiel
c. Conférences
d. Formations médicales continues (accréditées)
e. Autre: ____________________

Par quel organisme :_____________________

☐ Publication dans un journal scientifique
☐ Auprès d'une femme ou d'une fille que j'ai reçue en consultation
☐ Média (TV, radio, podcast, film..)
☐ Réseaux sociaux

☐ Informations personnelles
☐ Je ne me souviens pas
☐ Autre (merci de préciser) : ………………………………………..

4. Avez-vous déjà accompagné une femme ou une fille ayant subi une excision?

☐ Non (➔ question 5)
☐ Oui
☐ Je ne suis pas sûr.e

4.1 Si oui, combien de fois dans les 12 derniers mois (environ)?

☐ 0
☐ 1 à 5
☐ 6 à 10
☐ Entre 11 et 49
☐ Plus de 50
☐ Difficile à évaluer

5. Avez-vous déjà accompagné une femme ou une fille à risque de subir une excision?

☐ Non (➔ question 6)
☐ Oui
☐ Je ne suis pas sûr.e

5.1 Si oui, combien de fois dans les 12 derniers mois (environ)?

☐ 0
☐ 1 à 5
☐ 6 à 10
☐ Entre 11 et 49
☐ Plus de 50
☐ Difficile à évaluer

6. Vous a-t-on déjà demandé de pratiquer une excision sur une enfant mineure? (pour le personnel médical)

☐ Non jamais
☐ Oui très rarement (une ou deux fois)
☐ Oui plus souvent (plus que deux fois)
☐ Sans réponse

7. Vous a-t-on déjà demandé de pratiquer une réinfibulation après un accouchement? (pour les gynécos et sages-femmes)

☐ Non jamais
☐ Oui très rarement (une ou deux fois)
☐ Oui plus souvent (plus que deux fois)
☐ Sans réponse

Connaissances

8. D'après vous, combien de femmes excisées vivent en Belgique (approximativement)

☐ Moins de 6000
☐ 6 000
☐ 12 000
☐ 17 000
☐ 25 000
☐ 30 000
☐ Plus de 30 000
☐ Je ne sais pas

8.D'après vous, combien de femmes excisées vivent en France (approximativement)

☐ Moins de 60 000
☐ 60 000
☐ 120 000
☐ 450 000
☐ 980 000
☐ Plus de 980 000
☐ Je ne sais pas

9. L'OMS a décrit plusieurs types de mutilation génitale féminine (MGF). Combien en connaissez vous ?

Indiquez le nombre : __

9.1 Nommez et décrivez brièvement les différents types de mutilation génitale féminine repris dans la définition de l'OMS (utilisez une ligne par type)

a……………………………………………………………………
b……………………………………………………………………
c……………………………………………………………………
d……………………………………………………………………
e……………………………………………………………………
f……………………………………………………………………
g……………………………………………………………………

9.2 Vous sentez-vous capable de reconnaître les différents types selon la classification de l'OMS à l'examen clinique? (Pour le personnel médical et para)

☐ presque toujours
☐ avec plus ou moins de certitude
☐ avec peu de certitude
☐ pas du tout
☐ je ne sais pas, je n'ai jamais eu l'occasion de faire un tel examen

10. Abordez-vous le sujet de l'excision lors de votre pratique? (plusieurs réponses possibles)

☐ Non, jamais (➔ question 11)
☐ Oui, mais seulement si la femme/la famille en parle d'abord
☐ Oui mais seulement si je suspecte un risque d'excision chez l'enfant
☐ Oui, systématiquement quand une femme ou une fille est originaire d'un pays qui pratique (ou susceptible de pratiquer) l'excision
☐ Oui systématiquement sauf si la femme est accompagnée par un/des proche(s)
☐ Oui pour une autre raison, merci de préciser :…………………………………………….

10.1 Si oui, comment abordez-vous le sujet lors de la consultation ? (plusieurs réponses possibles)
☐ J'utilise une carte présentant les prévalences des MGF par pays
☐ Je remets des flyers ou livrets d'information
☐ Je lui montre des dessins avec les différents types d'excision et lui demande si elle connait le type de son excision.
☐ J'utilise des outils type vulve en silicone
☐ Je demande l'aide d'un.e collègue
☐ Je m'organise pour me trouver un moment seul.e avec la femme en faisant sortir si besoin les enfants, le mari ou d'autres accompagnants
☐ J'aborde le sujet spontanément et lui fournis des adresses afin de référer la femme si elle le souhaite
☐ Je lui demande si elle connait la loi en Belgique sur le sujet

☐ Autre (merci de préciser): ……………………………………………………………

11. Quels sont les facteurs de risque d'excision à repérer lors d'une consultation en présence d'un ou des deux parents et d'une petite fille ? (Plusieurs réponses possibles)

☐ La prévalence dans le pays d'origine des parents
☐ La prévalence dans le pays où le voyage est prévu
☐ Le groupe ethnique auquel elle appartient (groupe qui pratique les MGF)
☐ Le fait que la mère ou les sœurs soient excisées
☐ Les plans de voyage dans le pays d'origine, principalement si l'enfant est envoyée sans les parents

☐ La mauvaise qualité des échanges ou interactions avec les parents pendant la consultation
☐ La mauvaise compréhension d'une des langues nationales (barrière linguistique)
☐ Les signes extérieurs religieux
☐ Le fait de résider en Belgique depuis moins d'un an
☐ Quand dans une fratrie ce ne sont que les filles qui voyagent
☐ Quand les voyages sont prévus pour une longue période
☐ Le port d'habits traditionnels
☐ Le titre de séjour
☐ Autre (merci de préciser): ……………………………………………………………………

12. **Au cours d'une consultation vous apprenez que l'enfant qui est devant vous a été récemment excisée, que faites-vous?** (plusieurs réponses possibles)

☐ Je réalise un examen clinique (cette modalité n'apparait que pour le personnel médical)

☐ Je cherche s'il y a un.e soignant.e à proximité pour réaliser un examen clinique

☐ Je fais un signalement au procureur du Roi / de la République
☐ J'appelle la police

☐ Je rédige une « information préoccupante » pour la cellule de recueil des informations préoccupantes

☐ J'appelle le 112 pour une ambulance
☐ J'informe les parents que je vais réaliser un signalement
☐ J'écris une lettre au médecin généraliste ou au pédiatre pour l'informer afin qu'il fasse le signalement
☐ J'en parle avec les parents pour comprendre dans quelles conditions cette excision a été pratiquée
☐ Je rappelle aux parents que l'excision est passible de poursuites pénales
☐ J'explique aux parents les complications auxquelles cela l'expose
☐ Je me renseigne pour savoir s'il y a d'autres enfants à risque
☐ Je réfère les parents vers un centre de référence pour confirmation
☐ Je ne suis pas à l'aise avec le sujet et préfère ne pas en parler au risque de mal faire
☐ J'appelle une association spécialisée pour avoir des conseils sur la conduite à tenir (si oui, laquelle : ……………………………)
☐ Autre (merci de préciser):………………………………………………………

13. Concernant les MGF, comment estimez-vous le niveau de vos compétences?
☐ Très bien
☐ Bon

☐ Moyen
☐ Insuffisant
☐ Très insuffisant

14. Êtes-vous demandeur.s.e de suivre une formation ou d'une remise à niveau dans ce domaine?
☐ Non (➔ Question 13)
☐ Oui
☐ Je ne sais pas, pas sûr.e

14.1 Si oui, sur quels aspects la formation devrait porter :
☐ Aspects juridiques
☐ Aspects relationnels (interaction avec les usager.e.ss, attitudes)
☐ Accompagnement social
☐ Identification des MGF cliniquement
☐ Identification des enfants à risque de MGF
☐ Prise en charge si découverte d'une excision récente
☐ Prise en charge obstétricale
☐ Prise en charge sexologique
☐ Prise en charge chirurgicale
☐ Prévention de l'excision auprès d'enfants
☐ Intégration de la question des MGF dans l'éducation à la sexualité l'EVRAS (Education à la Vie Relationnelle, Affective et Sexuelle)
☐ Autre (merci de préciser) : ……………………………………………………………………

Etudes de cas

Pour toutes les professions

15. Vous venez de recevoir un appel d'une enseignante pour une jeune fille mauritanienne de 16 ans. Cette jeune fille est très stressée depuis deux mois, ses résultats scolaires sont en chute libre. Elle s'est confiée à son enseignante. Elle a très peur car les grandes vacances se rapprochent et elle est supposée voyager en Mauritanie où son futur mari choisi par ses parents l'attend. Elle a entendu que toutes les filles dans la famille sont excisées, elle a très peur. Que faites-vous? Plusieurs réponses possibles

☐ Je signale ce cas au procureur du Roi / de la République
☐ Je fais un signalement à la CRIP (cellule de recueil des informations préoccupantes)

☐ J'appelle la police
☐ Je contacte les parents pour discuter avec eux
☐ J'appelle la fille sur son GSM portable pour avoir sa version des faits

☐ Je demande à l'enseignante d'organiser une rencontre à l'école entre la jeune fille et une association spécialisée (mariage forcé et excision) pour confirmer le niveau de risque et être conseillé.e sur la marche à suivre

☐ Je contacte l'aide à la jeunesse

☐ Je demande conseil à un.e collègue

☐ Je cherche des options sur internet parce que je ne sais pas quoi faire

☐ J'appelle une association spécialisée pour avoir des conseils sur la conduite à tenir (si oui laquelle : ………………………………………………………….)

☐ Autre (merci de préciser:……………………………………………….)

Pour les professions médicales.

16. Vous êtes en consultation avec une mère et ses deux filles âgées respectivement de 3 et 11 ans. Elle vous annonce qu'un voyage au Mali est prochainement prévu pour visiter la famille durant 2 mois. Au cours de l'entretien, la grande soeur dit que la petite va être « coupée » durant les vacances. La mère vous rassure et vous explique que l'excision n'est plus pratiquée dans sa communauté. Que faites-vous? (plusieurs réponses possibles)

☐ J'informe la mère que l'excision est un crime en Belgique/France

☐ Je montre une carte des prévalences à la mère, lui montre le Mali et lui demande son avis sur cette pratique

☐ J'informe la mère des complications liées à l'excision

☐ J'essaye de savoir si la mère et la fille sont concernées par l'excision et si elles souhaitent un accompagnement

☐ Je crois la mère et je passe à un autre sujet

☐ Je demande à parler avec la fille aînée seule pour l'écouter

☐ Si après la discussion je sens que la mère est conciliante et que le voyage n'est pas prévu tout de suite, je fais signer les parents un certificat sur l'honneur qui stipulent qu'ils s'engagent à ne pas exciser l'enfant et qu'ils sont informés des peines potentiellement encourues

☐ J'appelle le médecin généraliste ou le pédiatre pour qu'il examine l'enfant avant et après le voyage

☐ Si le voyage est très proche et que la discussion avec la mère est bloquée, je réalise un signalement au procureur du Roi / de la République

☐ J'appelle une association spécialisée pour avoir des conseils sur la conduite à tenir (si oui laquelle : ……………………………………………………………..)

☐ Autre (merci de préciser):………………………………………………

Pour les professions médicales

17. Vous êtes en consultation avec une jeune femme et vous êtes amené.e à aborder le sujet de

l'excision. La jeune femme se tait, fond en larmes, puis parle sans croiser votre regard, sans s'arrêter, raconte une experience avec une gynécologue qui lui avait dit qu'elle était excisée mais qui ensuite est partie à la retraite et une deuxième qui ne lui en a jamais reparlé alors qu'elle y pense tous les jours, qu'elle fait des cauchemars, qu'elle entend les cris, les pleurs du jour de son excision… elle ne s'arrête pas, pleure continuellement et semble très angoissée. Que faites-vous? Plusieurs réponses possibles

☐ Je suis démuni.e et je sors du bureau pour chercher un.e collègue
☐ Je lui mets une main sur l'épaule et je l'invite à respirer tranquillement
☐ J'essaye de la ramener dans le moment présent, en l'appelant pas son prénom et en lui rappelant qu'elle est dans mon bureau en Belgique/France en sécurité
☐ Je me rends disponible et à l'écoute
☐ Je suis touché.e par ce qu'elle traverse et je lui en témoigne
☐ Je la fais sortir du bureau et la fais asseoir dans le couloir et je préviens la psychologue
☐ Je l'adresse aux urgences et fais une transmission au psychiatre de garde
☐ Je l'oriente vers un centre de référence (si oui, lequel…………………………………..)
☐ J'évalue l'impact et la prise en charge possible
☐ Je ne lui parle pas du risque pour ses filles, cela peut attendre

☐ Autre (merci de préciser): ……………………………………………………………………

18. **Vous rencontrez une femme guinéenne de 35 ans qui a 3 enfants et qui parle de reconstruction du clitoris. Elle a vu un reportage à la télé et elle dit que cela va résoudre tous ses problèmes car elle n'a pas de désir pour son mari. En l'écoutant, vous apprenez qu'elle a été mariée de force à 15 ans, que son mari a 20 ans de plus qu'elle, et qu'elle a été frappée plusieurs fois devant les enfants. Plusieurs réponses possibles**

☐ Je lui dis que ce sont des histoires personnelles et intimes qui ne sont pas de mon ressort
☐ Je l'informe qu'en Belgique/France il est possible de se séparer de son mari, et je lui donne les coordonnées d'un service de prise en charge des violences conjugales (si oui lequel : ………………………………)
☐ Je la réfère vers un centre multidisciplinaire qui propose la reconstruction du clitoris (si oui, lequel………………………………………………)
☐ Je la réfère vers une sexologue
☐ J'évoque le risque pour ses filles
☐ Je lui dis que je ne suis pas compétent.e pour répondre à cette question
☐ Je lui dis qu'elle est très courageuse de me parler de ce qu'elle vit à la maison
☐ Autre (merci de préciser) : ………………………………………

Pour les médecins généralistes ou gynécologues, les infirmières ou les sages-femmes

19. Vous voyez pour la première fois en consultation prénatale une jeune femme somalienne de 22 ans qui vient d'arriver en Belgique/France. C'est sa première grossesse, elle est à 32 SA. A l'examen clinique, vous voyez que sa vulve est complètement fermée par une infibulation (type III). Elle ne parle que quelques mots de français. La patiente est très timide et déjà très embarrassée par l'examen clinique, elle a mis son foulard sur ses yeux pendant l'examen tellement elle était gênée. Que faites-vous? Plusieurs réponses possibles

☐ Comme les paramètres vitaux de la mère et du bébé sont bons, je continue la consultation sans autre explication

☐ J'essaye de lui faire comprendre qu'elle aura une césarienne, comme sa vulve est complètement cousue, l'accouchement voie basse est impossible

☐ Je la réfère vers un.e collègue qui a plus d'expérience que moi sur les MGF pour qu'il/elle décide de la conduite à tenir

☐ Je cherche quelqu'un qui peut traduire pour elle et lui expliquer la situation

☐ Je préfère prendre un nouveau RDV avec une traductrice formée pour lui expliquer la situation

☐ Je me sens démuni.e, je ne sais pas quoi faire.

☐ Après avoir demandé son consentement, je prends une photo de sa vulve pour montrer à un.e collègue et avoir un deuxième avis.

☐ Je la rassure en lui expliquant qu'il est possible de pratiquer une désinfibulation avant l'accouchement afin d'éviter les complications

☐ Autre (merci de préciser):………………….. ……………………………………………

Pour les travailleurs sociaux et travailleuses sociales

20. Vous animez une école de devoir au CPAS. Une jeune fille de 13 ans qui fréquente votre école de devoir vient vous voir à la fin d'une séance. Elle se confie à vous : elle dit avoir subi une-excision enfant et elle pense que sa mère envisage de partir avec sa petite sœur de quatre ans au pays d'origine pour l'exciser. Que faites-vous? (Plusieurs réponses possibles)

Vous travaillez au sein d'une école. A la fin de la journée, une jeune fille se confie à vous : elle dit avoir subi une excision enfant et elle pense que sa mère envisage de partir avec sa petite sœur de quatre ans au pays d'origine pour l'exciser. Que faites-vous? (Plusieurs réponses possibles)

☐ Cette situation me dépasse complètement, je ne sais pas quoi faire. Je lui dis que je me renseigne et qu'on en reparlera plus tard.

☐ Je dis à la jeune de ne pas s'inquiéter qu'il y a des lois contre l'excision dans presque tous les pays, que cela ne peut plus arriver en 2021.

☐ Je vérifie la prévalence de taux d'excision dans son pays d'origine.

☐ Je demande à la jeune quand le voyage à lieu pour me donner une idée de l'urgence

☐ J'essaye de discuter avec la jeune fille sur la problématique de l'excision et ce qu'elle a vécu et j'évalue en premier lieu si elle a besoin de soins physiques ou psychologique avant de parler de sa petite sœur.

☐ Je prends directement contact avec les services d'aide à la jeunesse pour les informer de la situation

☐ Je contacte la police.

- ☐ J'appelle une association spécialisée pour avoir des conseils sur la conduite à tenir (si oui laquelle : ……………………………………………………………)
- ☐ J'invite la mère à un entretien sans sa fille afin d'avoir plus d'information.
  Si oui :
  - o Si après la discussion je sens que la mère est conciliante et que le voyage n'est pas prévu tout de suite, je fais signer les parents un certificat sur l'honneur qui stipule qu'ils s'engagent à ne pas exciser l'enfant et qu'ils sont informés des peines potentiellement encourues
  - o Je réfère directement la maman vers une association spécialisée pour qu'ils puissent la sensibiliser et lui parler directement car je ne me sens pas capable de donner cette information
  - o Si la mère semble pour l'excision, je demande à voir le papa (s'il vit en Belgique) pour connaître sa position
  - o Si le voyage est très proche et que la discussion avec la mère est bloquée, je réalise un signalement au procureur du Roi / de la République.

Pour les travailleurs sociaux et travailleuses sociales

21. Vous êtes travailleur.se social.e pour une ILA (Initiative Locale d'Accueil) ou dans un centre d'accueil collectif de demandeurs d'asile. Une femme originaire de Somalie et sa fille de 2 ans sont transférées dans votre ILA/votre centre d'accueil par le centre d'arrivée au Petit Château. Elle vous explique lors d'un entretien social qu'elle a fui pour des problèmes politiques. Son mari est a été tué par El Sheebab (groupe djihadiste).
Vous êtes travailleur.se social.e dans un centre d'accueil de demandeurs d'asile. Une femme originaire de Somalie et sa fille de 2 ans sont transférées dans votre votre centre d'accueil. Elle vous explique lors d'un entretien social qu'elle a fui pour des problèmes politiques. Son mari est a été tué par El Sheebab (groupe djihadiste) .

Partie 1 de la question. Elle ne parle pas d'excision dans son entretien. Que faites-vous ?

- ☐ J'aborde la problématique de l'excision avec la femme en utilisant la carte du monde des prévalences pour savoir si elle est concernée
- ☐ J'attends que la femme me parle de l'excision. Je n'aborde pas ce sujet si elle ne fait pas le premier pas. Je ne veux pas remuer de mauvais souvenir.
- ☐ Je m'assure d'abord de la présence d'une traductrice formée avant d'aborder la question de l'excision.
- ☐ Je lui demande si sa fille a déjà subi la pratique ou pas.

Partie 2 de la question. La femme me dit au cours de l'entretien qu'elle a été excisée et que sa fille ne l'a pas été comme dans sa communauté on le fait plus tard.

- ☐ J'oriente la mère vers un.e médecin formé.e pour qu'elle puisse avoir un certificat médical avec le type d'excision et une prise en charge des éventuelles complications médicales
- ☐ J'oriente également la fille vers un.e médecin formé.e pour qu'elle puisse avoir un certificat médical de non excision
- ☐ La fille n'a pas besoin de certificat médical comme elle n'a pas été excisée
- ☐ Je propose à la femme de se rendre dans une association spécialisée sur les MGF pour suivre des activités de groupe (si oui, laquelle ………………… ……………..)
- ☐ J'identifie un probable syndrome de stress post-traumatique chez la maman et la réfère vers un.e psychologue.

- ☐ J'informe sur la loi interdisant l'excision en Belgique.
- ☐ J'explique qu'en Belgique, les MGF sont considérés comme une violence basée sur le genre, qui peut donner droit à une protection nationale parce qu'on en a été victime et/ou pour s'en protéger (pour sa fille).
- ☐ Je ne me sens pas du tout formé.e pour aborder cette thématique et préfère ne pas en parler.
- ☐ J'ai été formé.e sur la question et j'utilise la check-list et la trajectoire MGF et Asile pour savoir quoi faire.

Information socio-démographique

22. Vous êtes:

☐ Un homme

☐ Une femme

☐ Autre:

23. Quel âge avez-vous?

☐ 20-39 ans

☐ 40-54 ans

☐ 55 ans ou plus

24. Depuis combien d'années exercez-vous cette profession? ___ ___
