# Supplementary material for: Knowledge of female genital cutting among health and social care professionals in Francophone Belgium: A cross-sectional survey
Source: PLOS Glob Public Health. 2024 Jul 18;4(7):e0002225. doi: 10.1371/journal.pgph.0002225 (PMC11257331; doi:10.1371/journal.pgph.0002225)
Supplement: Attachment — Submitted filename: Review comments for HCPs and SCPs Knowledge on FGC in Belgium 19.9.23.docx [file pgph.0002225.s003.docx]

**Knowledge of Female Genital Cutting among health and social care professionals in Francophone Belgium: A cross-sectional survey**

The authors present findings of the research study titled “Knowledge of Female Genital Cutting among health and social care professionals in Francophone Belgium: A cross-sectional survey”.

This is an important paper that adds to the evidence on the knowledge level of health professionals and social care professionals in Belgium. The findings can be a strong basis for advocacy for structured training of the professionals to take lead in FGM prevention and response in Belgium and Europe where a lot of migrants with FGM related complications live. However, I find some gaps that need to be addressed before the paper can be published as is. The following are minor comments towards the manuscript.

**Abstract**

1. The authors should not start sentences with numbers
2. Results section: “Those who had already provided care for FGM/C were more numerous to know that there were 4 types (52%). This statement is confusing it need to be clarified.

**Methods**

3. It will be helpful to provide a description of the study setting to help the readers conceptualize the findings and conclusion
4. The authors need to provide a description of the health system of the francophone Belgium to help conceptualize the findings
5. What was the inclusion criteria for the study participants?
6. What setting was the health care worker should have been working in for them to have participated in the study?
7. The subsection on statistics should be replaced with data analyses
8. Did the authors assess the knowledge about re-infibulation among the participants?
9. Did the authors ask where (source) the health and social professionals could have acquired the knowledge on FGM from?
