# Supplementary material for: Knowledge of female genital cutting among health and social care professionals in Francophone Belgium: A cross-sectional survey
Source: PLOS Glob Public Health. 2024 Jul 18;4(7):e0002225. doi: 10.1371/journal.pgph.0002225 (PMC11257331; doi:10.1371/journal.pgph.0002225)
Supplement: Attachment — Submitted filename: response letter PLOS global public health RESUB.docx [file pgph.0002225.s004.docx]

RESPONSE LETTER TO PLOS GLOBAL HEALTH

16 November 2023

Dear Editor,

We have revised the manuscript following the reviewers’ helpful comments and suggestions. The responses to their suggestions are below.

Many thanks

Best wishes

Dr Sarah O’Neill

Journal Requirements:

1. Please provide additional details regarding participant consent. In the ethics statement in the Methods and online submission information, please ensure that you have specified (1) whether consent was informed and (2) what type you obtained (for instance, written or verbal, and if verbal, how it was documented and witnessed). If your study included minors, state whether you obtained consent from parents or guardians. If the need for consent was waived by the ethics committee, please include this information.

*The survey targeted adults about their professional practice. They received an invitation to participate via email through their professional organisation (see page 9). The first page of the online survey explained the objectives and the background of the study, stating that no personal identifiers of participants were recorded, and that the study was completely anonymous. Before the beginning the survey, all participants had to confirm their informed consent to participate by clicking to continue with the survey. No minors participated in the study.*

2. If you are reporting a retrospective study of medical records or archived samples, please ensure that you have discussed whether all data were fully anonymized before you accessed them and/or whether the IRB or ethics committee waived the requirement for informed consent. If patients provided informed written consent to have data from their medical records used in research, please include this information.

N/A

3. Please provide separate figure files in .tif or .eps format only and remove any figures embedded in your manuscript file. Please also ensure all files are under our size limit of 10MB.

Ok

For more information about figure files please see our guidelines:

<https://journals.plos.org/globalpublichealth/s/figures> 
[https://journals.plos.org/globalpublichealth/s/figures#loc-file-requirement](https://journals.plos.org/globalpublichealth/s/figures#loc-file-requirement)

4. We noticed that you used "unpublished" in the manuscript. We do not allow these references, as the PLOS data access policy requires that all data be either published with the manuscript or made available in a publicly accessible database. Please amend the supplementary material to include the referenced data or remove the references.

The reference has been taken out of the text, we merely refer to the author of the MA dissertation and the results.

5. We have noticed that you have uploaded Supporting Information files, but you have not included a list of legends. Please add a full list of legends for your Supporting Information files after the references list.

*Ok a full list of legends can be found after the reference list*

**Reviewer Comments to the Author**

Reviewer #1: Thank you for the opportunity to review this paper. The issue of female genital cutting or female genital mutilation is an important area to explore and I commend the authors on this work. It is clear that with global migration more women who have experienced FGM are moving to other regions and so knowledge of FGM is important in all countries.

In the Abstract, I suggest writing a clear aim. This should also be in the end of the Background. I always like to see a statement that is “the aim of this study was to ….”

*Thanks for the suggestion. The last sentence now reads: “The study-objective was to gain a better understanding of the knowledge and practices of professionals working in the area of health in Francophone Belgium where no previous KAP has been done.”*

In terms of the sample, I couldn't work out why infectious diseases specialists and internal medicine specialists were surveyed. I was not sure of the relevance for those two disciplines but without a clear aim I cannot be sure.

*In Belgium travel clinic consultations are done by professionals with a background in infectious disease or internal medicine. Travel clinics are also the places where people go for vaccination and to prepare for a trip to tropical countries, including Africa. When migrants residing in Belgium plan to return to a country where FGC is practised their uncut daughters are perceived to be ‘at risk’ of undergoing FGM/C. Health professionals working in travel clinics are in a good position to addresses FGM/C as a potential risk and remind the parents/guardians that performing FGM/C back home is illegal and that if their daughters are cut on holiday, parents/guardians residing in Belgium are liable to prosecution and a prison sentence if found guilty. Our study is not the first to include professionals at travel clinics, an interesting study was published by Tantet et al 2018 in France on infectious diseases specialists’ the knowledge of FGC.*

The studies conducted in one part of Belgium, but this is a study in four countries Belgium, Canada, France and Sweden. I could not work out why it was only in one part of Belgium (Francophone Belgium) if it's a national program. Why wasn't in the other parts of the country, especially when a lot of the data presented in study setting is about the numbers of women in the whole of Belgium, who are at risk of have this procedure or have had this procedure.

*Regrettably this was due to a funding issue. The funding for the Belgian part of the study was allocated by the FNRS/FRSM dedicated to research in Francophone Belgium only. We have added a sentence to make this clearer in the Methods section on page 6. We would have preferred for the survey to cover health and social care professionals across the whole of Belgium. Two previous KAP studies had already been undertaken in the Dutch-speaking part of Belgium but not the Francophone part. The Francophone Belgian data will eventually be compared to the data from the French team.*

Only five gynaecologists responded to the survey which feels like a significant problem.

*A total of 166 medical doctors participated in the survey. As the invitations were sent via professional organisations, we were not able to send reminders to participate and have no control over the final numbers. The strength of this study however is to assess knowledge of FGM/C among other health and social care professionals who care for women on a day to day basis.*

Some of the phrases are not clear. In paragraph two on page 10 I don't understand what ‘having consciously accompanied a woman or a girl with FGM’ means. Does that mean caring for a woman. Later on that paragraph is another phrase – ‘ the most numerous to have accompanied women or girls with FGM’. I'm assuming that again, is about caring for so perhaps a bit of attention needs to be placed on the phraseology.

*Accompanied has been replaced by ‘provided care or support’. Apologies for the French translation.*

I was interested in the model of care that these women experienced in Francophone Belgium. Did they have continuity of care? Did they see midwives in the hospital in the community? Were they mostly seeing midwives or doctors?

*We have added the following sentence to the study setting section: “Belgium has a national social security system for all residents with an added provision for undocumented persons. This means that all people have access to care.” This is followed by a reference refers to the SPF official website outlining social security services. Depending on their needs, women can be seen by midwives, gynaecologists or any other medical professional. Social services are mostly provided by social workers. If conventional (not private) professionals’ services are sought these services are entirely covered by national social security.*

In the beginning of the discussion, I was hoping to an overarching summary of the results before we delve into the different areas.

*There is a summary of the results each section of the discussion. This is split into: Rate of exposure to FGM/C by profession; other determinants of exposure; knowledge and attitude; knowledge and prevalence; capacity of identify and categorize; requests for FGM/C and re-infibulation ; discomfort with addressing the topic.*

As a non European, I found use of Flemish/Flanders, Francophone Belgium terms and naming the different regions quite confusing. For those living in Europe, it's probably very straightforward, but I found it quite difficult to follow.

*Yes, the Belgium state is not homogenous in terms of population, Flanders is the Dutch-speaking region and Wallonia is the French-speaking region. I have tried to clarify this further in the study setting section pages 6-7.*

There are a number of limitations in the study but the authors do not detail these. The small number of participants in the study especially gynaecologists, is a significant issue. Recall bias is also a limitation. I think the survey probably attracted respondents who knew more about FGM so then the sample is quite biased.

*Thank you for these helpful suggestions. I have added the following to the limitations section: “Recall bias is always a risk when asking health-professionals questions on the numbers of patients they have seen. It is also likely that those who already had some knowledge of the practice and were interested in the topic participated in the survey whereas those with no particular interest in the subject did not. These are the pitfalls that all surveys need to take into consideration.”*

I was surprised that there did not seem to be question about how to actually care for these women. The focus seemed to be on identification rather than actual care?

*The reason for this is that we were targeting such a broad variety of health and social care professionals who care for affected women in such different ways. A second part of the questionnaire does address different ways of caring for and responding to specific situations among different kinds of health and social care professionals. However, this will be published later on.*

There are significant issues with the tables for tables. There are too many - nine tables and they look like they've just been taken the cross-tab output from SPSS or whatever statistical program and put into the tables. They're all formatted differently.

*We have reformatted the tables and cut two table and the figure. Thank you for the suggestion which we think will improve clarity.*

The references also were quite messy with uppercase titles and some of the journal abbreviations are not clear (eg Ref 12).

*Thank you for this helpful suggestion. We have corrected some of the mistakes in the references including reference 12. As for the abbreviations, the reference manager follows the PLOS recommended style Vancouver. This is done automatically following Vancouver style.*

Some minor issues
In the abstract the acronym FGM/C is used, but it's not written out.
The same issue for KAP in the Background.

*The word count is limited for the abstract, which is why we prefer to use the acronyms. However, the words are fully written out in the title of the article.*

Reviewer #2: Knowledge of Female Genital Cutting among health and social care professionals in Francophone Belgium: A cross-sectional survey
The authors present findings of the research study titled “Knowledge of Female Genital Cutting among health and social care professionals in Francophone Belgium: A cross-sectional survey”.
This is an important paper that adds to the evidence on the knowledge level of health professionals and social care professionals in Belgium. The findings can be a strong basis for advocacy for structured training of the professionals to take lead in FGM prevention and response in Belgium and Europe where a lot of migrants with FGM related complications live.

**Abstract**

1. The authors should not start sentences with numbers

Ok

2. Results section: “Those who had already provided care for FGM/C were more numerous to know that there were 4 types (52%). This statement is confusing it need to be clarified.

I have changed the sentence to: Those who had already provided care for women with FGM/C were more numerous to know that there were four types (52%).

**Methods**

3. It will be helpful to provide a description of the study setting to help the readers conceptualize the findings and conclusion

*Thank you for this suggestion. More details on the linguistic geography of Belgium has been added to the study setting section page 6 and 7.*

4. The authors need to provide a description of the health system of the francophone Belgium to help conceptualize the findings

*This has also been added to page 6 and 7.*

5. What was the inclusion criteria for the study participants?

The inclusion criteria are implicitly stated on page 7: “The specific focus of this questionnaire was that it should target healthcare as well as social care professionals. Although this study was designed to give an overview of FGM/C related knowledge, competences, attitudes and the need to undertake further training among professionals in Francophone Belgium, … “

We believe that this statement is explicit enough, adding inclusion criteria would appear repetitive.

6. What setting was the health care worker should have been working in for them to have participated in the study?

*The study participants were contacted through their professional organisation, which are listed on page 9: Union professionel des Sages-femmes Belges (UPSfB) and Association Francophone des sages-femmes catholiques (AFSFC) for midwifery, Union des villes et des communes de Wallonie (UVCW) for social workers in the Belgian welfare centre, Federal Agency for Reception of Asylum Seekers (Fedasil) of the Brussels and Wallonie region for social workers working with asylum seekers; Office de la Naissance et de l’enfance (ONE) the national agency for health and social care workers providing antenatal and postnatal care services;*  Société Scientifique de Médecine Générale *(SSMG) for generalist practitioners;*

*Centre Hospitalier Chrétien (CHC) for « infectious disease specialists and internal medicine »****;*** *Groupement Belge des Pédiatres de langue Française* ***(****GBPF) for paediatricians; Groupement des Gynécologues Obstétriciens de Langue Française de Belgique (GGOLFB)*

7. The subsection on statistics should be replaced with data analyses

*Ok the sub-heading has been changed to data analysis.*

8. Did the authors assess the knowledge about re-infibulation among the participants?

*Medical professionals were asked if they had received requests for re-infibulation. Please see result section page 11 for details. No other questions assessing knowledge on re-infibulation were asked.*

9. Did the authors ask where (source) the health and social professionals could have acquired the knowledge on FGM from?

*Yes, there were questions on sources of information on FGM/C. However, we have not included the responses in this article. We are considering publishing the results in another article.*
