# Supplementary material for: Knowledge of female genital cutting among health and social care professionals in Francophone Belgium: A cross-sectional survey
Source: PLOS Glob Public Health. 2024 Jul 18;4(7):e0002225. doi: 10.1371/journal.pgph.0002225 (PMC11257331; doi:10.1371/journal.pgph.0002225)
Supplement: Attachment — Submitted filename: PLOS response to Reviewer May 2024.doc [file pgph.0002225.s005.doc]

Reviewer #1: Thank you for addressing all my comments.
My only suggestion is the tables, They do look a lot better however I still think they could be combined and improved. I will leave it to the Editor to decide.

*I thank the reviewer for the useful suggestion. We have modified table 7.*

Reviewer #2: Knowledge of Female Genital Cutting among health and social care professionals in Francophone Belgium: A cross-sectional survey
The authors present findings of the research study titled “Knowledge of Female Genital Cutting among health and social care professionals in Francophone Belgium: A cross-sectional survey”.
This is an important paper that adds to the evidence on the knowledge level of health professionals and social care professionals in Belgium. The findings can be a strong basis for advocacy for structured training of the professionals to take lead in FGM prevention and response in Belgium and Europe where a lot of migrants with FGM related complications live.

*Thanks*

The comments identified in the previous review have been addressed comprehensively.
The following TWO very minor comments need to be addressed as well
Abstract: Most of the comments were compressively addressed
1. Results section: “Those who had already provided care for FGM/C were more numerous to know that there were 4 types (52%). This statement is not clear, and I modify it to read “Those who had already provided care to women with FGM/C were more likely to know that there were 4 types (52%)”. Kindly adopt it to make it clear.

*No probability analysis has been done, we therefore do not know of they were “more likely” to know the 4 types. Our analysis show that they were more numerous to know that there were 4 types. We therefore prefer to stick to our original formulation.*

2. All the comments in the methods sections have been addressed comprehensively.

*OK*

Results
3. The authors should remove the % symbol in the in texts with the tables but include it on the top row of each table to avoid repetition and redundancy.

*The % symbol is in the headings of tables 1-6 which reflect the responses of the same numbers of participants indicated in N in the headings too. Table 7 is different in that the number of respondents per profession differs on each line. The numbers of research participants who responded to those questions are indicated in N on each line. It is therefore preferable to keep the % sign in each field for clarity, so that no confusion arises with regards to numbers of participants.*

*The layout of the table has been modified for clarity and looks better. I hope that the reviewer is satisfied with these changes.*
